# Analysis of serum insulin-like growth factor-1, fibroblast growth factor 23, and Klotho levels in girls with rapidly progressive central precocious puberty

**DOI:** 10.1007/s00431-023-05174-y

**Published:** 2023-08-29

**Authors:** Yuping Liu, Yaying Cheng, Meng Sun, Xiaojing Hao, Mengmeng Li

**Affiliations:** Department of Pediatrics, Hebei General Hospital, 348 Heping West Road, Xinhua District, Shijiazhuang, Hebei China

**Keywords:** Rapidly progressive central precocious puberty, IGF-1, FGF23, Klotho

## Abstract

To study the levels of serum insulin-like growth factor 1 (IGF-1), fibroblast growth factor 23 (FGF23), and Klotho, and to study their relationship with girls with rapidly progressive central precocious puberty (RP-CPP). This is a cross-sectional study on the progression rate of central precocious puberty in girls, who complained of breast development before the age of 8 years and were followed between June 2021 and June 2022. At the same time, 28 healthy girls less than 8 years old who had not started puberty were recruited as the control group. The physical examination and laboratory evaluation of each group was completed. Only patients with CPP received pelvic ultrasound examination and bone age test. Bone age index (BAI), basal LH levels (BLH), basal LH levels/basal FSH levels (BFSH), peak LH (PLH)/peak FSH (PFSH), IGF-1, Klotho, FGF23, and ovarian volume in the RP-CPP group were higher than those in slowly progressive CPP (SP-CPP) group. In the RP-CPP group, IGF-1 was correlated with Klotho, FGF23, and BLH; Klotho was correlated with FGF23 and BLH; FGF23 was correlated with BLH.

*Conclusion*: The BLH, FGF23, Klotho, and IGF-1 have a certain correlation with RP-CPP, which may play an important role in the speed of girls’ sexual development.

**Table:** 

**What is Known:** *• The association between IGF-1 and RP-CPP.*	
**What is New:** *• We found the association between FGF23, Klotho and RP-CPP.*

**What is Known:**

*• The association between IGF-1 and RP-CPP.*

**What is New:**

*• We found the association between FGF23, Klotho and RP-CPP.*

## Introduction

Central precocious puberty (CPP) refers to the emergence of secondary sexual characteristics caused by early activation of the hypothalamic-pituitary-gonadal axis (HPGA) in girls before the age of 8 years [1]. CPP can be divided into slowly progressive central precocious puberty (SP-CPP) and rapidly progressive central precocious puberty (RP-CPP). SP-CPP refers to the slow progress of sexual development and bone age, and the linear growth remains at the corresponding percentile, and RP-CPP refers to the rapid sexual development process, from one Tanner stage to the next stage within 6 months [2], often accompanied by impaired final adult height (FAH), premature menarche, and subsequent psychological and social problems. Therefore, these children need early identification and treatment.

Puberty development is regulated by the growth hormone (GH)- insulin-like growth factor 1 (IGF-1) axis and HPGA. Activation of the HPG axis promoted the secretion of GH and IGF-1, and estrogen further promoted the secretion of GH, resulting in increased concentrations and enhanced effects of IGF-1 in the growth plate [3]. Studies have found that IGF-1 stimulates a peptidergic pathway within the medial basal hypothalamus (MBH) to eliminate the inhibition of gonadotropin-releasing hormone (GnRH) secretion before puberty, and IGF-1 seems to play a role in activating and enhancing GnRH secretion during puberty [4]. Therefore, we propose that IGF-1 is the link between linear growth and reproductive development in humans. The researchers found that as the onset of puberty approached, the level of IGF-1 increased, which induced the release of GnRH before puberty, and can make the onset of puberty earlier [4]. One study found that IGF-1 levels were higher in CPP girls than in healthy controls [5]. He et al. found that not only the serum IGF-1 level of girls in the ICPP group was higher than that in the control group, but also the serum IGF-1 level was positively correlated with bone age [6]. Moreover, a clinical study found that the level of IGF-1 in serum can be used as a diagnostic indicator of CPP [7].

Klotho is an anti-aging factor associated with female reproductive endocrine, and its abnormal expression may affect reproductive function. The level of Klotho is affected by the state of pubertal development. The serum Klotho level of children after puberty initiation is higher than that before puberty initiation [8]. Studies have shown that Klotho is a regulator of the GH-IGF-1 axis, which promotes GH secretion and is positively correlated with IGF-1 levels [9]. FGF23 is an important regulatory factor of bone minerals, which, together with Klotho, is involved in the regulation of the homeostasis metabolism of bone minerals in humans [10]. A study found that the level of c-terminal FGF23 in healthy children is related to age, with the highest levels in infancy and adolescence [11]. Meanwhile, Klotho is an indispensable part of the activation of the FGF23 signaling pathway [12]. CPP girls have the problem of advanced bone age. There is no study has found that FGF23, Klotho, and RP-CPP link of the girls.

At present, the identification of RP-CPP mainly relies on follow-up monitoring of growth and bone age. During the follow-up, the linear growth potential of some children has been impaired, so this study aims to explore whether there is a certain correlation between Klotho, FGF23, IGF-1, and RP-CPP girls.

## Method

### Study population

From June 2021 and June 2022, 210 girls under 8 years old with CPP who complained of premature thelarche were recruited from Hebei General Hospital. CPP inclusion criteria [13]: (1) girls had premature thelarche before 8 years old; (2) the annual linear growth rate was higher than that of normal children, which refers to bone age growth/age growth > 1; (3) bone age is more than one year ahead of chronological age; (4) pelvic ultrasound showed that ovarian volume ≥ 1 mL, the critical value of uterine volume was 3.4~4 mL, and several follicles with diameter > 4 mm could be seen in the ovary; (5) GnRH stimulation test showed that PLH > 5 IU/L, PLH/PFSH > 0.6. After 6 months of follow-up, CPP girls were finally divided into the RP-CPP group (30 cases) and SP-CPP group (31 cases) according to the speed of sexual development. The inclusion criteria of the RP-CPP group were as follows [14]: (1) rapid sexual development, Tanner stage progressed to the next stage within 6 months; (2) height increased more than 4 cm in 6 months; (3) bone age progressed over 1 year during follow-up. The inclusion criteria of the SP-CPP group [14]: (1) no progression of Tanner stage during follow-up and (2) linear growth and actual age remained at the corresponding percentile. At the same time, 28 healthy girls under 8 years old who did physical examinations in the Hebei General Hospital at the same time were recruited as the control group. Children who were lost to follow-up initiated GnRHa treatment in advance, other subsequent precocious puberty, other diseases, and recent use of antibiotics and steroid hormones were excluded. We excluded 149 girls in the CPP group because of loss of follow-up, early initiation of GnRHa treatment, other diseases, pituitary magnetic resonance abnormalities, and recent use of antibiotics and steroid hormones. This study was approved by the Ethics Committee of Hebei General Hospital and conducted by the requirements of the Declaration of Helsinki. All participants obtained informed consent.

### Examinations

Girls in the CPP group and control group: Tanner stage, height, and weight were assessed by the same pediatric physician, and body mass index (BMI), BMI standard deviation score (BMI-SDS), and height SDS were calculated. All children with CPP were required to complete blood routine, liver and kidney function, thyroid function, LH, FSH, and estradiol (E2) measurements at 8 am at the first visit. At the same time, the GnRH stimulation test was performed to complete the determination of BLH, BFSH, PLH, and PFSH. All CPP patients underwent pelvic ultrasound, bone age, and pituitary MRI examination. Bone age was measured by the Greulich and Pyle atlas, and bone age index (BAI) was obtained by dividing bone age by chronological age (CA). FGF23 (Cusabio, China) and Klotho (4A Biotech, China) were determined by enzyme-linked immunosorbent assay (ELISA). The intra-assay coefficients of variations (CVs) of FGF23 were < 8%, and the inter-assay CVs were < 10%. The intra- and inter-assay CVs of Klotho was < 10%. IGF-1 (Snibe, China) was determined by chemical luminescence (Immulite 2000, Siemens), the intra-CVs ≤ 10%, and the inter-CVs ≤ 15%. Girls in the CPP group and the control group need to improve the determination of FGF23, Klotho, and IGF-1 levels.

### Statistical analysis

Statistical analysis was performed using the SPSS software 26.0. Measurement data with normal distribution were expressed as mean ± standard deviation, and a *t*-test was used to determine statistical differences. Measurement data with skewed distribution were expressed as M (*P*25, *P*75). The Wilcoxon rank-sum test was used for comparison between the two groups, and the Kruskal Wallis test was used for comparison between the three groups. Spearman correlation analysis was used to evaluate the correlation. *P* < 0.05 was considered statistically significant.

## Results

### Clinical characteristics in RP-CPP and SP-CPP

Sixty-one children were diagnosed with CPP, including 30 girls diagnosed with RP-CPP (mean age 7.37 ± 0.35 years) and 31 girls diagnosed with SP-CPP (mean age 7.30 ± 0.34 years). The general characteristics of both groups are shown in Table 1. The BAI of the RP-CPP group was significantly higher than that of the SP-CPP group (*P* = 0.0001). In terms of sex hormone indicators, the levels of BLH, BLH/BFSH, and PLH/PFSH were also significantly different between the two groups (*P* < 0.05). The ovarian volume of the RP-CPP group was significantly larger than that of the SP-CPP group (*P* = 0.015).

**Table 1:** Clinical characteristics of RP-CPP and SP-CPP girls

	RP-CPP	SP-CPP	*P*-value
*n*	30	31	
CA, years	7.37 ± 0.35	7.30 ± 0.34	0.393
Height, cm	130.39 ± 4.05	128.97 ± 4.50	0.198
Height SDS	1.15 ± 0.57	0.96 ± 0.59	0.225
Weight, kg	32.68 ± 4.3	31.78 ± 5.2	0.463
BMI, kg/m^2^	16.56 ± 0.60	16.80 ± 0.80	0.191
BMI-SDS	0.37 ± 0.28	0.46 ± 0.24	0.208
BAI	1.17 ± 0.07	1.12 ± 0.04	0.000^a^
E2, pg/mL	51.19 (32.62, 63.65)	40.18 (33.56, 50.65)	0.282
Basal LH, IU/L	0.77 (0.62, 1.16)	0.32 (0.17, 0.49)	0.000^a^
Basal FSH, IU/L	3.6 (2.39, 4.85)	2.87 (2.1, 4.15)	0.105
LH/FSH, basal	0.24 ± 0.06	0.11 ± 0.03	0.000^a^
Peak LH, IU/L	16.08 ± 6.63	14.03 ± 5.3	0.184
Peak FSH, IU/L	11.06 ± 3.1	11.96 ± 2.22	0.199
LH/FSH, peak	1.19 (1.02, 1.6)	1.17 (0.82, 1.37)	0.011^a^
Ovarian volume, mL	2.52 ± 0.8	2.04 ± 0.71	0.015^a^

*RP-CPP* rapidly progressive central precocious puberty, *SP-CPP* slowly progressive central precocious puberty, *n* number, *CA* chronological age, *BA* bone age, *BMI* body mass index, *SDS* standard deviation score, *BAI* bone age index, *E2* estradiol, *LH* luteinizing hormone, *FSH* follicle-stimulating hormone

^a^Indicates statistical significance (*P* < 0.05)

### Comparison of IGF-1, FGF23, and Klotho levels among all subjects

There were significant differences in serum IGF-1, Klotho, and FGF23 levels among the RP-CPP group, SP-CPP group, and control group (*P* = 0.0001). Girls in the RP-CPP group had higher serum levels of IGF-1, Klotho, and FGF23 than those in the SP-CPP and control groups (Table 2).

**Table 2:** Comparison of IGF-1, FGF23, and Klotho levels among all subjects

	RP-CPP	SP-CPP	Control group	*P*-value
*n*	30	31	28	
IGF-1, ng/mL	375.3 (281.98, 416.2)^a,b^	269.4 (230.3, 324.5)	207.45 (182.65, 246.285)	0.0001
Klotho, pg/mL	1950.81 (1875.65, 2078.37)^a,b^	1773.84 (1731.96, 1835.48)	1657.74 (1498.75, 1713.25)	0.0001
FGF23 pg/mL	42.30 (38.24, 48.80)^b,c^	38.73 (34.73, 42.66)	28.75 (27.21, 33.55)	0.0001

*IGF-1* insulin-like growth factor 1, *FGF23* fibroblast growth factor 23, *RP-CPP* rapidly progressive central precocious puberty, *SP-CPP* slowly progressive central precocious puberty, *n* number

^a^Indicates statistical significance *P* < 0. 0001, compared with the SP-CPP group

^b^Indicates statistical significance *P* < 0. 0001, compared with the control group

^c^Indicates statistical significance *P* < 0. 01, compared with the SP-CPP group

### The correlation analysis of RP-CPP predictors

In the RP-CPP group, the serum Klotho level was significantly correlated with IGF-1, estradiol, and LH (*r* = 0.668, *r* = 0.380, *r* = 0.538, *P* < 0.05); the serum FGF23 level was significantly correlated with IGF-1, LH base value, and age (*r* = 0.64, *r* = 0.376, *r* = 0.741, *P* < 0.05, respectively). The specific correlation coefficients are shown in Table 3.

**Table 3:** Relationship among IGF-1, Klotho, and FGF23 and CPP parameters in the RP-CPP group

	Klotho		FGF23		IGF-1	
	*r*	*P*-value	*r*	*P*-value	*r*	*P*-value
IGF-1	0.668	0.000^a^	0.64	0.000^a^	-	-
E2	0.380	0.038^a^	0.334	0.071	0.512	0.004^a^
Basal LH	0.538	0.002^a^	0.376	0.041^a^	0.511	0.004^a^
LH/FSH, basal	0.034	0.858	0.245	0.191	−0.195	0.302
LH/FSH, peak	0.338	0.068	0.199	0.532	0.259	0.167
Height SDS	0.051	0.787	0.197	0.297	0.25	0.182
BMI SDS	0.099	0.604	−0.156	0.409	−0.008	0.966
BAI	−0.11	0.562	−0.288	0.123	−0.158	0.404
CA	0.313	0.093	0.741	0.000^a^	0.768	0.000^a^
Ovarian volume	0.119	0.533	0.293	0.116	0.445	0.014^a^

*FGF23* fibroblast growth factor 23, *E2* estradiol, *LH* luteinizing hormone, *FSH* follicle-stimulating hormone, *BMI* body mass index, *SDS* standard deviation score, *BAI* bone age index, *CA* chronological age

^a^Indicates statistical significance (*P* < 0.05); IGF-1, insulin-like growth factor 1

## Discussion

The purpose of this study was to explore the relationship between FGF23 and Klotho and RP-CPP, in order to provide a new perspective for the diagnosis and treatment of RP-CPP. Most precocious puberty girls with premature thelarche aged 7–8 years olds are SP-CPP without treatment [15], while children with RP-CPP usually have FAH damage due to delayed treatment, so physicians should pay more attention to RP-CPP. The FAH in children with RP-CPP treated with GnRHa has a greater benefit in the lower age group [16]. Meanwhile, it is impossible to distinguish early RP-CPP and SP-CPP only by anthropometric measurements [15].

The BA of girls in the RP-CPP group was significantly higher than that in the SP-CPP group. In this study, BAI was used as a more intuitive indicator, when BAI > 1, it means that BA is ahead of the CA, if BAI < 1, it means that BA is behind CA [1]. This study found that the BAI of the RP-CPP group was significantly higher than that of the SP-CPP group. When ovarian volume > 2 mL, it was significantly correlated with the occurrence of RP-CPP [17], which was consistent with the results of this study. LH and FSH together maintain normal reproductive function in women. E2 is an important hormone that promotes the development of female internal and external reproductive organs and maintains secondary sexual characteristics. The elevation of the basal LH fully reflects the initiation of the HPG axis. In this study, when the basic value of LH is greater than 0.55 IU/L, it is necessary to be alert to the occurrence of RP-CPP. Calcaterra et al. [17] found that when the basic value of LH > 0.2 IU/L, it is closely related to the occurrence of RP-CPP.

IGF-1 is a polypeptide hormone mainly secreted by hepatocytes, it participates in a variety of physiological activities in the body, such as promoting the repair of injured peripheral nerves [18], increasing the density of bone minerals to prevent fractures [19], and regulating brain development. The GH-IGF-1 axis can stimulate the proliferation of chondrocytes on the growth plate, which plays a decisive role in regulating human linear growth [20]. Some scholars have found that IGF-1 and its receptors are expressed in all neuroendocrine cells in the rat anterior pituitary, and the protein expression level is the highest in growth hormone cells and gonadotropin cells [21]. Studies have found that IGF-1 can eliminate the inhibition of GnRH secretion before puberty by stimulating the peptide energy pathway in the medial basal hypothalamus, thereby further increasing the release of GnRH [4]. Animal studies show that IGF-I enhances GnRH-stimulated LH release without changing the number of GnRH receptors in cattle [22]; at the same time, IGF-I enhances luteinizing hormone release from rat [23]. It has long been found that serum IGF-I levels were not only an independent predictor of early menarche, but IGF-I can also regulate the reproductive system through a wide range of effects on the hypothalamus, pituitary gland, and ovary [24]. There was a positive correlation between serum IGF-1 value and serum basal LH value [25]. This study also found that in the RP-CPP group, the IGF-1 was positively correlated with the classical CPP parameter LH, which was consistent with the results of Escagedo et al. [3] The study confirmed that the levels of serum LH and IGF-1 in CPP girls were higher than those in the control group [7]. This study found that the IGF-1 of girls in the RP-CPP group was higher than that in the SP-CPP group. Biro et al. found that IGF-1 concentration was higher in girls with earlier onset of puberty and menarche [26]. On the one hand, IGF-1 plays a role in inducing the proliferation of chondrocytes and osteoblasts to regulate linear growth in the process of human puberty; on the other hand, it can also regulate the HPG axis by regulating the central nervous system.

FGF23 is a hormone mainly secreted by bone cells and is important for maintaining bone health and mineral homeostasis [27]. An animal experiment confirmed that mice with FGF23 gene ablation showed short life, kyphosis, and hypogonadism [28]. Leptin is an important adipocytokine secreted by white adipose tissue, which plays an important role in pubertal development. When the nutritional status of the body meets the reproductive conditions, leptin can be used as a starting factor to activate GnRH neurosecretion [29]. Leptin can also promote the pituitary secretion of LH and FSH. The leptin level of CPP girls was significantly higher than that of the control group [30]. Leptin can also directly stimulate FGF23 secretion in osteocytes [31], and leptin levels are positively correlated with FGF23 levels [32]. This study found that the FGF23 level of CPP girls was significantly higher than that of the control group, and the FGF23 level of RP-CPP girls was the highest. This suggests that FGF23 may be involved in the initiation of puberty and may also be related to the speed of puberty progression. This study also found that FGF23 level was positively correlated with basal LH, IGF-1, and CA. Dagmar et al. demonstrated that FGF23 is positively correlated with age, and its levels are highest in infancy and adolescence, which is consistent with the results of the present study [11].

The Klotho gene is an aging suppressor gene. FGF23 cannot exert its biological activity without Klotho. Klotho is highly expressed in mature mouse germ cells in an animal experiment [33]. This study found that the Klotho level of CPP girls was significantly higher than that of the control group, and the Klotho level of RP-CPP girls was the highest. Klotho can be used as an early warning indicator of RP-CPP. This indicates that the Klotho level may be related to the speed of puberty progression, and a higher Klotho level indicates faster CPP progression. Klotho is moderately expressed in the ovarian and pituitary tissues of mice [34]. This study also found that the Klotho level was positively correlated with basal LH, but no correlation with age. Gkentzi et al. [8] also confirmed that the Klotho level was not related to age, but related to sexual maturity. This suggests that FGF23 and Klotho may be involved in the initiation of puberty. At the same time, this study found that the Klotho level was positively correlated with the IGF-1 level. This conclusion has been widely confirmed. Studies have found that IGF-1 stimulates the secretion of Klotho [35]. IGF-1 directly promoted Klotho shedding, while IGF-1 and Klotho concentrations were positively correlated [36], which is consistent with the results of this study. Klotho is a direct regulator of GH secretion [37]. Sex hormones are very important for bone metabolism, and bone mineral quality is related to sex hormone levels. Meanwhile, related enzymes and receptors of sex hormones are also expressed in bone tissues [38], which also explains that children with CPP not only have rapid sexual development but also have impaired linear growth potential. Therefore, it can be considered that Klotho, FGF23, and IGF1 may be closely linked through a complex mechanism and play an important role in the regulation of human puberty and growth and development. Its specific mode of action and related mechanisms need to be further studied.

## Limitations

This is a single-center study that requires further large-scale, multi-center studies on more patients to confirm our findings. Due to the limitations of cross-sectional design, this study could not clarify the causal relationship between serum FGF23 and Klotho levels and RP-CPP. Prospective studies should be conducted to summarize these findings and make them more comprehensive. The specific mechanism of the role of FGF23-Klotho axis in puberty is not clear and needs further study.

## Summary

This study showed that BAI was significantly higher in the RP-CPP group than in the SP-CPP group, suggesting that RP-CPP children may have accelerated bone age and impaired linear growth potential in the early stage of the disease. BAI has a certain guiding significance for evaluating the speed of sexual development in CPP girls. The levels of serum IGF-1, FGF23, and Klotho in the RP-CPP group were significantly higher than those in the SP-CPP group and control group, suggesting that serum IGF-1, FGF23, and Klotho play a certain role in the initiation of puberty in girls. Klotho, FGF23, and IGF-1 are closely related to the occurrence and development of RP-CPP.

## Data Availability

The data that support the findings of this study are available from the corresponding author upon reasonable request.

## References

[1] Latronico AC, Brito VN, Carel JC (2016). Causes, diagnosis, and treatment of central precocious puberty. Lancet Diabetes Endocrinol.

[2] Carel JC, Léger J (2008). Clinical practice. Precocious puberty. N Engl J Med.

[3] Escagedo PD, Deal CL, Dwyer AA, Hauschild M (2021). Insulin-like growth factor 1, but not insulin-like growth factor-binding protein 3, predicts central precocious puberty in girls 6–8 years old: a retrospective study. Horm Res Paediatr.

[4] Dees WL, Hiney JK, Srivastava VK (2021). IGF-1 Influences gonadotropin-releasing hormone regulation of puberty. Neuroendocrinology.

[5] Sørensen K, Aksglaede L, Petersen JH, Andersson AM, Juul A (2012). Serum IGF1 and insulin levels in girls with normal and precocious puberty. Eur J Endocrinol.

[6] He J, Kang Y, Zheng L (2018) Serum levels of LH, IGF-1 and leptin in girls with idiopathic central precocious puberty (ICPP) and the correlations with the development of ICPP. Minerva Pediatr. 10.23736/S0026-4946.18.05069-7 10.23736/S2724-5276.18.05069-7 30037185

[7] He J, Kang Y, Zheng L (2023) Correlation of serum levels of LH, IGF-1 and leptin in girls with the development of idiopathic central precocious puberty. Minerva Pediatr (Torino) 75(3):381–386. 10.23736/S2724-5276.18.05069-7 10.23736/S2724-5276.18.05069-7 30037185

[8] Gkentzi D, Efthymiadou A, Kritikou D, Chrysis D (2014). Fibroblast growth factor 23 and Klotho serum levels in healthy children. Bone.

[9] Shahmoon S, Rubinfeld H, Wolf I (2014). The aging suppressor klotho: a potential regulator of growth hormone secretion. Am J Physiol Endocrinol Metab.

[10] Razzaque MS (2009). The FGF23-Klotho axis: endocrine regulation of phosphate homeostasis. Nat Rev Endocrinol.

[11] Fischer DC, Mischek A, Wolf S (2012). Paediatric reference values for the C-terminal fragment of fibroblast-growth factor-23, sclerostin, bone-specific alkaline phosphatase and isoform 5b of tartrate-resistant acid phosphatase. Ann Clin Biochem.

[12] Abraham CR, Li A (2022) Aging-suppressor Klotho: prospects in diagnostics and therapeutics. Ageing Res Rev 82:101766. 10.1016/j.arr.2022.101766 10.1016/j.arr.2022.101766 36283617

[13] Cheuiche AV, da Silveira LG, de Paula LCP, Lucena IRS, Silveiro SP (2021). Diagnosis and management of precocious sexual maturation: an updated review. Eur J Pediatr.

[14] Calcaterra V, Sampaolo P, Klersy C (2009). Utility of breast ultrasonography in the diagnostic work-up of precocious puberty and proposal of a prognostic index for identifying girls with rapidly progressive central precocious puberty. Ultrasound Obstet Gynecol.

[15] Chen Y, Liu J (2021). Do most 7- to 8-year-old girls with early puberty require extensive investigation and treatment?. J Pediatr Adolesc Gynecol.

[16] Li P, Li Y, Yang CL (2014) Gonadotropin releasing hormone agonist treatment to increase final stature in children with precocious puberty: a meta-analysis. Medicine (Baltimore) 93(27):e260. 10.1097/MD.0000000000000260 10.1097/MD.0000000000000260 PMC4602779 25501098

[17] Calcaterra V, Klersy C, Vinci F (2020). Rapid progressive central precocious puberty: diagnostic and predictive value of basal sex hormone levels and pelvic ultrasound. J Pediatr Endocrinol Metab.

[18] Hanwright PJ, Qiu C, Rath J et al (2022) Sustained IGF-1 delivery ameliorates effects of chronic denervation and improves functional recovery after peripheral nerve injury and repair. Biomaterials 280:121244. 10.1016/j.biomaterials.2021.121244 10.1016/j.biomaterials.2021.121244 34794826

[19] Yuan S, Wan ZH, Cheng SL, Michaëlsson K, Larsson SC (2021). Insulin-like growth factor-1, bone mineral density, and fracture: a Mendelian randomization study. J Clin Endocrinol Metab.

[20] Börjesson AE, Lagerquist MK, Liu C (2010). The role of estrogen receptor α in growth plate cartilage for longitudinal bone growth. J Bone Miner Res.

[21] Eppler E, Jevdjovic T, Maake C, Reinecke M (2007). Insulin-like growth factor I (IGF-I) and its receptor (IGF-1R) in the rat anterior pituitary. Eur J Neurosci.

[22] Hashizume T, Kumahara A, Fujino M, Okada K (2002). Insulin-like growth factor I enhances gonadotropin-releasing hormone-stimulated luteinizing hormone release from bovine anterior pituitary cells. Anim Reprod Sci.

[23] Soldani R, Cagnacci A, Yen SS (1994). Insulin, insulin-like growth factor I (IGF-I) and IGF-II enhance basal and gonadotrophin-releasing hormone-stimulated luteinizing hormone release from rat anterior pituitary cells in vitro. Eur J Endocrinol.

[24] Daftary SS, Gore AC (2005). IGF-1 in the brain as a regulator of reproductive neuroendocrine function. Exp Biol Med (Maywood).

[25] Ouyang L, Yang F (2022). Combined diagnostic value of insulin-like growth factor-1, insulin-like growth factor binding protein-3, and baseline luteinizing hormone levels for central precocious puberty in girls. J Pediatr Endocrinol Metab.

[26] Biro FM, Huang B, Wasserman H, Gordon CM, Pinney SM (2021). Pubertal growth, IGF-1, and windows of susceptibility: puberty and future breast cancer risk. J Adolesc Health.

[27] Li X (2019). The FGF metabolic axis. Front Med.

[28] Shimada T, Kakitani M, Yamazaki Y (2004). Targeted ablation of Fgf23 demonstrates an essential physiological role of FGF23 in phosphate and vitamin D metabolism. J Clin Invest.

[29] Shi L, Jiang Z, Zhang L (2022). Childhood obesity and central precocious puberty. Front Endocrinol (Lausanne).

[30] Zurita-Cruz JN, Villasís-Keever MA, Manuel-Apolinar L et al (2021) Altered cardiometabolic profile in girls with central precocious puberty and adipokines: a propensity score matching analysis. Cytokine 148:155660. 10.1016/j.cyto.2021.155660 10.1016/j.cyto.2021.155660 34334260

[31] Białka-Kosiec A, Orszulak D, Gawlik A, Drosdzol-Cop A (2022). The relationship between the level of vitamin D, leptin and FGF23 in girls and young women with polycystic ovary syndrome. Front Endocrinol (Lausanne).

[32] Ratsma DMA, Zillikens MC, van der Eerden BCJ (2021) Upstream regulators of fibroblast growth factor 23. Front Endocrinol (Lausanne) 12:588096. 10.3389/fendo.2021.588096 10.3389/fendo.2021.588096 PMC7952762 33716961

[33] Li SA, Watanabe M, Yamada H, Nagai A, Kinuta M, Takei K (2004). Immunohistochemical localization of Klotho protein in brain, kidney, and reproductive organs of mice. Cell Struct Funct.

[34] Olauson H, Mencke R, Hillebrands JL, Larsson TE (2017). Tissue expression and source of circulating αKlotho. Bone.

[35] Caicedo D, Díaz O, Devesa P, Devesa J (2018) Growth hormone (GH) and cardiovascular system. Int J Mol Sci 19(1). 10.3390/ijms19010290 10.3390/ijms19010290 PMC5796235 29346331

[36] Rubinek T, Shahmoon S, Shabtay-Orbach A (2016). Klotho response to treatment with growth hormone and the role of IGF-I as a mediator. Metabolism.

[37] Rubinek T, Wolf I, Modan-Moses D (2016) The longevity hormone Klotho is a new player in the interaction of the growth hormone/insulin-like growth factor 1 axis. Pediatr Endocrinol Rev 14(1). 10.17458/PER.2016.RWM.LongevityHormoneKlotho 10.17458/PER.2016.RWM.LongevityHormoneKlotho 28508612

[38] Holmlund-Suila E, Viljakainen H, Ljunggren Ö, Hytinantti T, Andersson S, Mäkitie O (2016). Fibroblast growth factor 23 concentrations reflect sex differences in mineral metabolism and growth in early infancy. Horm Res Paediatr.

